# *Cryptosporidium* impacts epithelial turnover and is resistant to induced death of the host cell

**DOI:** 10.1128/mbio.01720-24

**Published:** 2024-07-12

**Authors:** Bethan A. Wallbank, Ryan D. Pardy, Igor E. Brodsky, Christopher A. Hunter, Boris Striepen

**Affiliations:** 1 Department of Pathobiology, School of Veterinary Medicine, University of Pennsylvania, Philadelphia, Pennsylvania, USA; Stony Brook University, Stony Brook, New York, USA

**Keywords:** *Cryptosporidium*, apicomplexan parasites, epithelial cells, gastrointestinal infection, diarrhea, apoptosis, malnutrition, cell proliferation

## Abstract

Infection with the apicomplexan parasite *Cryptosporidium* is a leading cause of diarrheal disease. Cryptosporidiosis is of particular importance in infants and shows a strong association with malnutrition, both as a risk factor and as a consequence. *Cryptosporidium* invades and replicates within the small intestine epithelial cells. This is a highly dynamic tissue that is developmentally stratified along the villus axis. New cells emerge from a stem cell niche in the crypt and differentiate into mature epithelial cells while moving toward the villus tip, where they are ultimately shed. Here, we studied the impact of *Cryptosporidium* infection on this dynamic architecture. Tracing DNA synthesis in pulse-chase experiments *in vivo,* we quantified the genesis and migration of epithelial cells along the villus. We found proliferation and epithelial migration to be elevated in response to *Cryptosporidium* infection. Infection also resulted in significant cell loss documented by imaging and molecular assays. Consistent with these observations, single-cell RNA sequencing of infected intestines showed a gain of young and a loss of mature cells. Interestingly, enhanced epithelial cell loss was not a function of enhanced apoptosis of infected cells. To the contrary, *Cryptosporidium*-infected cells were less likely to be apoptotic than bystanders, and experiments in tissue culture demonstrated that infection provided enhanced resistance to chemically induced apoptosis to the host but not bystander cells. Overall, this study suggests that *Cryptosporidium* may modulate cell apoptosis and documents pronounced changes in tissue homeostasis due to parasite infection, which may contribute to its long-term impact on the developmental and nutritional state of children.

**IMPORTANCE:**

The intestine must balance its roles in digestion and nutrient absorption with the maintenance of an effective barrier to colonization and breach by numerous potential pathogens. An important component of this balance is its constant turnover, which is modulated by a gain of cells due to proliferation and loss due to death or extrusion. Here, we report that *Cryptosporidium* infection changes the dynamics of this process increasing both gain and loss of enterocytes speeding up the villus elevator. This leads to a much more immature epithelium and a reduction of the number of those cells typically found toward the villus apex best equipped to take up key nutrients including carbohydrates and lipids. These changes in the cellular architecture and physiology of the small intestine may be linked to the profound association between cryptosporidiosis and malnutrition.

## INTRODUCTION

*Cryptosporidium* is a parasite of mucosal surfaces, and depending on the host species, it can infect the epithelia of the gastro-intestinal or respiratory tracts. Human infection, caused most prominently by *Cryptosporidium hominis* and *Cryptosporidium parvum*, is localized to the small intestine and results in watery diarrheal disease that lasts 1–2 weeks in immunocompetent hosts. Infection can be persistent and fatal in immunocompromised individuals and infants, with the most significant disease burden falling on young children in resource-limited settings (1, 2). Children who experience cryptosporidiosis are highly susceptible to malnutrition and growth stunting, extending the impact of the acute infection into later life (3). There are no vaccines, and the only FDA-approved drug available for the treatment of cryptosporidiosis is ineffective in malnourished and immunocompromised individuals (4). Infection with *Cryptosporidium* is spread among hosts via the oocyst stage, most commonly in contaminated drinking and recreational water (5, 6). Following oocyst ingestion, motile sporozoite stages are released in the intestinal lumen and invade the apical surface of intestinal epithelial cells (IECs). *Cryptosporidium* resides just under the membrane of the host cell, surrounded by a parasitophorous vacuole, with a complex interface that separates the parasite from the host cell cytoplasm. *Cryptosporidium* parasites do not breach the epithelial layer but instead remain at the apical surface of the IEC, facing into the lumen of the gut. Here, they undergo three rounds of asexual replication before forming sexual stages, which through fertilization produce new infectious oocysts that can be shed into the feces to infect new hosts or reinfect the same host within the intestinal lumen (7).

The epithelium of the small intestine, in which the parasite replicates, is a highly structured yet dynamic tissue consisting of repeating units of villi containing absorptive enterocytes and proliferative crypts. The small intestinal epithelium undergoes rapid regeneration and turnover. IECs are formed in a stem cell niche at the base of the crypt, differentiate into the various epithelial cell types, and move up the villus axis. Old or damaged cells are then extruded at the top of the villus (8–10). This process takes 2–4 days in mice and 4–5 days in humans (11, 12). It is important that balance is maintained between the introduction of new cells and the shedding of older cells to sustain the absorptive and defensive capabilities of the mucosal barrier. When this balance is shifted due to acute damage or infection, the gut responds to maintain the equilibrium. For example, cells adjacent to sites of damage can alter their ultrastructure and migrate to the injury to restore the barrier. Additionally, stem cell proliferation and subsequent differentiation are similarly enhanced to replace the damaged epithelial cells (13–15). However, sustained damage or severe trauma, such as that experienced in inflammatory bowel disease, can overwhelm the system, resulting in a chronic imbalance between regeneration and death leading to a significant loss of absorptive function and barrier integrity (16, 17).

Post-mortem histological studies of humans and animals infected with *Cryptosporidium* have demonstrated significant pathological changes to the intestine. These included villus blunting and atrophy, crypt hyperplasia and branching, an increased mitotic rate, and epithelial dysplasia (18–22). While *Cryptosporidium* infection, thus, clearly impacts the global structure of the intestinal epithelium, the mechanism of this disruption remains poorly understood. Here, we used imaging-based and molecular assays to dissect the relationship between infection and IEC proliferation. We find infection results in significant increases in both cell proliferation and loss. Experimental perturbation of epithelial turnover increases parasite burden during infection, suggesting that this physiological response plays a part in parasite restriction. We analyze the association between cell death and parasite infection and find that *Cryptosporidium* infection confers enhanced resistance to apoptosis of the cells it infects.

## RESULTS

### Epithelial cell proliferation, migration, and loss are elevated during *Cryptosporidium* infection

**Fig 1:**
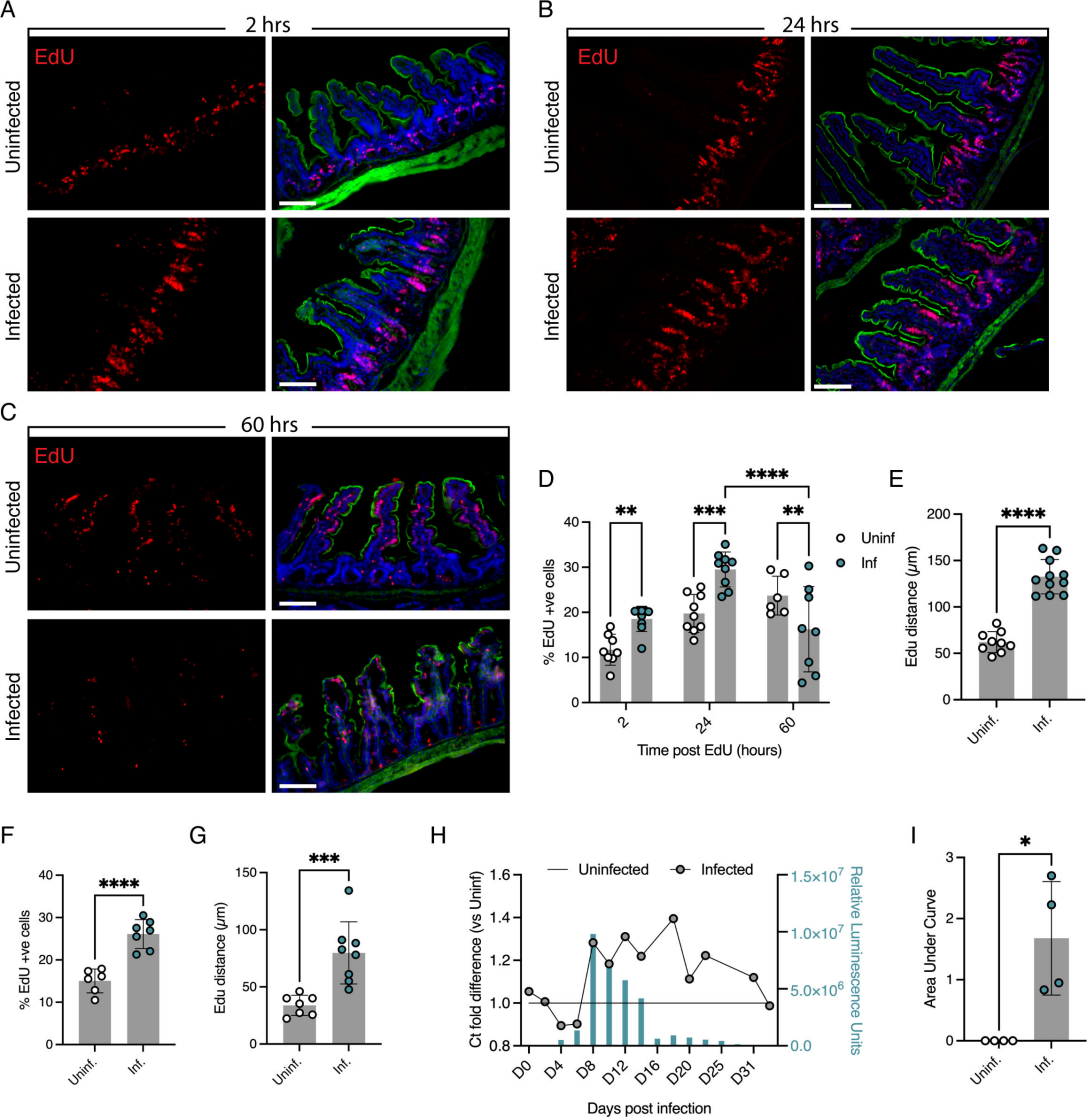
Epithelial cells are turned over faster with infection. (**A–C**) Micrographs of representative intestinal sections of frozen OCT-embedded tissues sampled 2 (**A**), 24 (**B**), and 60 (**C**) hours after EdU injection. Proliferating cells that have incorporated EdU into their nuclei are labeled with Alexa Fluor 594 dye (red). Phalloidin in green and Hoechst-stained IEC nuclei in blue. Scale bar, 100 µm. (**D**) Percentage of EdU-positive cells 2, 24, and 60 hours after EdU injection. Each data point represents a mouse and the mean of three images of a tissue section. Three to four mice were used in each of the four (2 and 24 hours) and two (60 hours) independent experiments. **P < 0.01, ***P < 0.001, and ****P < 0.0001, multiple comparisons two-way ANOVA, and error bars show SD. (**E**) Distance from top of crypt to the furthest EdU-labeled cell averaged across three images per mouse. Each data point represents a mouse and the mean of three images. Three to four mice in each of the three independent experiments. ****P < 0.0001, *t*-test. Error bars show SD. (**F**) Percentage of EdU-positive cells at 24 hours post-EdU injection of C57BL/6 mice. Each data point represents a mouse and the mean of two quantified images. Three to four mice in each of the three independent experiments. ****P < 0.0001, *t*-test. Error bars show SD. (**G**) Distance from top of crypt to furthest EdU-labeled cell in C57BL/6 mice. Data points represent individual mice and the mean of two images. Three to four mice in two independent experiments. ***P < 0.001, *t*-test. Error bars show SD. (**H**) Mouse genomic DNA (gDNA) extracted from feces and assessed by qPCR. Relative quantities shown as cycle threshold (Ct) fold difference comparing infected to uninfected. NanoLuc luciferase (nLuc) was measured in pooled feces from three mice per group and plotted as bars. A representative example of four independent experiments is shown here (all four experiments are shown in Fig. S1B). (**I**) Area under the curve of Ct fold differences from qPCR of fecal mouse gDNA. Each data point represents one independent experiment. Feces pooled from three mice per group per experiment, four independent experiments. *P < 0.05, Welch’s *t*-test. Error bars show SD.

To understand how infection impacts the turnover of the epithelium, we used an imaging-based approach to quantify the rate of turnover during infection and focused specifically on the ileum, the area of the small intestine where *Cryptosporidium* is most abundant (23). *Ifnγ^−/−^* mice were used as they support a more robust *C. parvum* infection when compared to immunocompetent C57BL/6 mice (24), and uninfected *Ifnγ^−/−^* mice served as controls. *Ifnγ^−/−^* mice were infected with 10,000 *C*. *parvum* oocysts and injected with 5-ethynyl-2-deoxyuridine (EdU) on day 8 of infection, when oocyst shedding peaks. EdU is incorporated into nascent DNA during synthesis, and incorporation is then visualized by reaction with a fluorescent dye azide by click chemistry (25). Due to the very brief bioavailability of EdU [<1 hour (26)], only proliferative cells that are undergoing DNA replication within this timeframe are labeled. Ileal sections were taken 2, 24, and 60 hours post-EdU injection and imaged (Fig. 1A through C). Images were segmented using Imaris software, and EdU-labeled nuclei were quantified as the percentage of total cell nuclei identified by labeling with the DNA dye Hoechst. Relative to uninfected mice, samples collected at 2 and 24 hours after injection from infected mice showed a 1.5- and 1.6-fold increase in EdU-positive cells, respectively (*P* < 0.01, *P* < 0.001; Fig. 1D). This indicated an increase in cell proliferation. In uninfected mice at 60 hours post-EdU injection, EdU-positive cells were localized near the tips of the villi, reflecting a homeostatic cell turnover time of 3 days (Fig. 1C). However, in infected samples, the percentage of EdU-positive cells was significantly lower when compared to both the uninfected control (*P* < 0.01) and the 24-hour infected time point (*P* < 0.0001) (Fig. 1D). This suggests that cell turnover time from crypt to villus tip was faster in infected mice and that cells were lost due to infection. Note that the baseline number of EdU-positive cells in uninfected mice also increases significantly between 2 and 24 hours (*P* = 0.0066) and 2 and 60 hours (*P* = 0.0002) (but not between 24 and 60). This likely reflects additional mitoses in a subset of EdU-labeled cells in the transit amplifying zone as they mature into the various intestinal epithelial cell types (27). A similar increase was noted in previous studies (25, 28, 29). To measure cell migration up the villus, we also quantified the distance between the top of the crypt and the most distal EdU-labeled nucleus. At 24 hours post-EdU injection, this distance was 2.2-fold greater in infected than in the uninfected mice (*P* < 0.0001; Fig. 1E), demonstrating that infection results in enhanced cell migration up the crypt–villus axis. Increases in cell proliferation and migration (*P* < 0.001; Fig. 1G) were also seen in C57BL/6 wild-type mice when infected with 100,000 oocysts of a mouse-adapted *C. parvum* strain (ma*Cp*) capable of infecting immunocompetent mice (23). We note that these differences were not the result of differential sampling along the ileum (Fig. S1A).

Our imaging-based assays suggested that cells were being lost from the villi at a greater rate during infection. To test this using a molecular approach, we quantified amounts of mouse gDNA in the fecal matter of uninfected and infected mice. qPCR was performed using primers against the mouse 18S ribosomal RNA locus (30), and fecal nLuc was used to simultaneously measure parasite burden (31), as parasites expressed the nLuc enzyme (Fig. 1H). The amount of mouse DNA shed with the feces in infected mice was significantly higher than that of uninfected mice (*P* < 0.05; Fig. 1I). Parasite burden peaked at day 8, which correlated with the peak of mouse gDNA in the feces (Fig. 1H; Fig. S1B). Interestingly, heightened cell loss extended past the drop in oocyst shedding before resolving to the level of uninfected mice. Whether elevated cell loss is due to parasite-mediated cell lysis or host-mediated cell death remains unknown. We conclude that *Cryptosporidium* infection significantly increases cell proliferation in the crypt, migration along the villus, and loss due to cell death or shedding.

### Elevated turnover of infected intestines is reflected in the epithelial transcriptome

**Fig 2:**
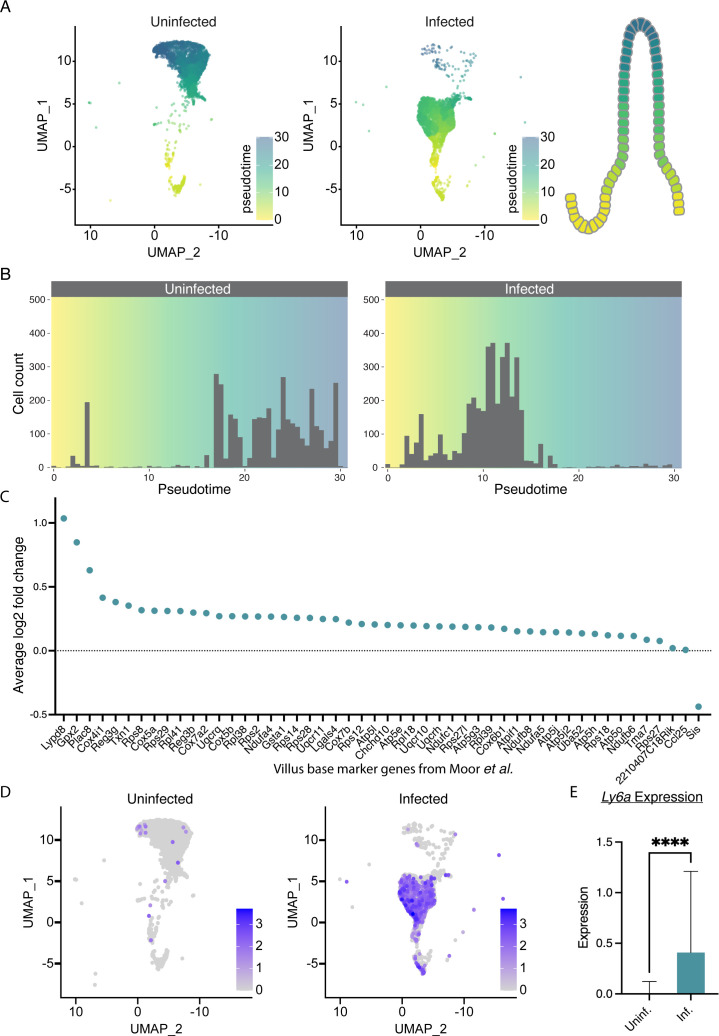
Single-cell RNA-seq of infected intestines reveals a more immature transcriptional state of the epithelium. (**A**) UMAP analysis of scRNA-seq of intestinal epithelial cells from uninfected and infected *Ifnγ^−/−^* mice. Pseudotime was projected onto the UMAP to reflect the epithelial axis of developmental maturation shown schematically for an ileal villus. Cells from two uninfected and two infected *Ifnγ^−/−^* mice were pooled. UMAP representations of data from 6,263 and 6,666 cells derived from uninfected and infected mice, respectively. (**B**) Histograms binning cells based on their pseudotime value from uninfected and infected mice from panel **A**. Note the shift to the left upon infection, reflecting a less mature developmental state. (**C**) Average log2 fold change due to infection of marker genes of the immature villus base (clusters 1 and 2 from reference 34). Note across the board increase with infection. (**D, E**) Heatmap of *Ly6a* gene expression overlayed on scRNA-seq UMAP (**D**) and plotted as average expression of all cells (**E**) from infected and uninfected intestines. Two pooled mice per sample. ****P < 0.0001, *t*-test. Error bars show SD.

We next asked how infection-induced epithelial changes impact the IEC transcriptome and drew upon a single-cell RNA sequencing (scRNA-seq) data set from our laboratories that compared transcriptomes in IECs of ma*Cp*-infected and uninfected *Ifnγ^−/−^* mice (32). Figure 2A shows uniform manifold approximation and projection (UMAP) plots for cells isolated from dissociated intestinal epithelia in the presence or absence of infection. Cell types were identified using transcriptional markers, and pseudotime analysis was used to computationally order cells based on their transcriptional progression through differentiation and development (33). As a result, low pseudotime values represent stem cells and “immature” epithelial cells, while increasing values indicate more differentiated cells located closer to the villus tip (Fig. 2A). In infected mice, we observed a marked increase in lower pseudotime values representing stem cell and villus base markers and a loss of mature, high-pseudotime cells (Fig. 2B). This shift could reflect an increase in stem cell proliferation and a loss of more mature cells from the villus tips. To confirm this observation of increased proportions of lower pseudotime cells, we plotted the log2 fold change in mRNA abundance for transcriptional markers of villus base and stem cells. As a reference, we used genes identified by Moor et al. as positional markers by laser capture of microdissected villi (34). Figure 2C shows that all but one of these markers displayed an increase in log2 fold change upon infection. This reflects a shift in the proportion of immature to mature cells likely though elevated turnover, consistent with our imaging-based findings. Infection may also reshape the stem cell niche through non-positional changes in the transcriptional program. Interestingly, we also observed a significant increase in Ly6a expression with infection (*P* < 0.0001; Fig. 2D and E), a surface protein commonly associated with cellular proliferation. Ly6a is a marker of proliferative, fetal-like, intestinal stem cells and is not widely expressed in the adult small intestine at homeostasis (35). Increased expression of Ly6a has previously been associated with intestinal wound healing through fetal-like reversion of crypt cells during colitis (36) and parasitic helminth infection (35). Interestingly, we also observed an increase in the expression of other proliferation-associated markers, such as epidermal growth factor receptor (Egfr) and Ki67 (37) (Fig. S2A and D). Overall, we find a marked change in the composition and gene expression of the epithelial layer to a more immature state during infection.

### Epithelial migration leads to parasite accumulation toward the villus tip

**Fig 3:**
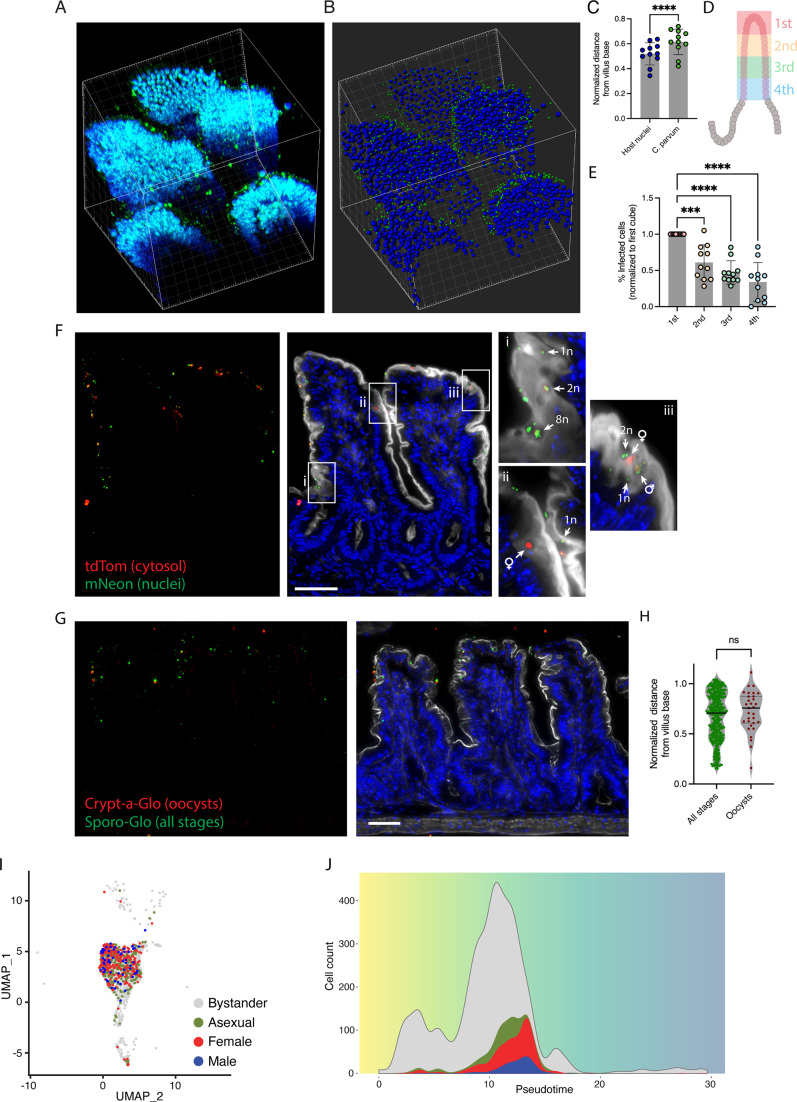
Imaging and sequencing analysis demonstrates a skewing of infected cells to the upper portion of the villus. (**A**) Z-stack and (**B**) segmentation-based volume of six villi rendered using Imaris from images of infected intestine taken by two-photon microscopy. Hoechst-stained IEC nuclei in blue and mNeon parasites in green. Grid square = 20 µm. (**C**) Mean distance of host nuclei and parasites from base of villus with top of stack normalized to 1. Each data point represents quantification of a stack from one mouse. Three to four mice for each of three independent experiments. *P < 0.0001, *t*-test. Error bars show SD. (**D**) Schematic of villus split into cubes along Z-plane for quantification. (**E**) Parasite distribution split into cubes according to panel **D**. Each data point represents quantification of a stack from one mouse. Three to four mice for each of three independent experiments. ***P < 0.001; ****P < 0.0001, multiple comparison one-way ANOVA. Error bars show SD. (**F**) Tissue section from *C. parvum* H2B-mNeon tdTom-infected intestine. Red is parasite cytosol, green is parasite nuclei, and blue is Hoechst-stained IEC nuclei. (i, ii, and iii) Insets from different positions along villus axis demonstrating no apparent stage-specific clustering. Arrows point to examples of 1, 2, and 8 nuclei (n) asexual parasites and male and female stages. Scale bar, 50 µm. (**G**) Representative micrograph of Sporo-Glo (green; all parasite stages) and Crypt-a-Glo (red and green co-staining; oocysts)-stained, *C. parvum*-infected, intestinal sections. Scale bar, 50 µm. (**H**) Quantification of normalized oocyst distance from the villus base from Crypt-a-Glo and Sporo-Glo dual-stained sections. Each data point represents one parasite, two villi quantified per image. Three mice in each of two independent experiments. No significant difference is seen between oocyst stages and all parasite stages, *t*-test. (I) UMAP of enterocytes from infected *Ifnγ^−/−^* mice. Infected cells are highlighted, and the parasite stage is indicated by color. Two pooled mice per sample. UMAP show data from 6,666 cells. (J) Cell counts from (**I**) plotted according to pseudotime [uninfected bystanders (gray), cells infected with asexual (green), female (red), or male (blue) parasites].

Next, we asked whether the observed increase in epithelial cell proliferation and migration affected the distribution of the parasites along the villus axis. Ileal tissue was taken from mice infected with *C. parvum* expressing the neon fluorescent protein, fixed, and imaged by multiphoton microscopy. To increase the depth of laser penetration through the tissue, the mice were perfused with phosphate-buffered saline (PBS) and formaldehyde, and the tissue was cleared using N-butyldiethanolamine and Triton (CUBIC-L) (38). The cleared intestine was opened longitudinally and imaged from the luminal surface, which generated Z-projections of the entire villi (Fig. 3A). Fluorescent parasites and Hoechst-stained host IEC nuclei were identified by segmentation using Imaris software (Fig. 3B), and their relative distribution along the villus axis was quantified. For each image, the distance of the host nuclei and the parasites from the base of the villus was quantified. The villus tip at the top of the Z-stack was normalized to 1. The mean position of the parasites was significantly higher up the villus than the mean position of epithelial nuclei demonstrating that the parasites are closer to the villus tip than the villus base (Fig. 3C). To gain a second more detailed breakdown of parasite position along the villus, the Z-stack was divided into four equal cubes along the villus axis, and each cube was scored for the percentage of infected host cells (Fig. 3D). Infected cells were most prevalent in the first cube at the top of the villus (Fig. 3E). We, therefore, conclude that while parasites can infect along the length of the villus, infected cells are preferentially found toward the villus tip.

*Cryptosporidium* undergoes three rounds of asexual replication before differentiating into distinct male and female stages (7). To assess whether the stages of the parasite life cycle are differentially distributed, we infected mice with parasites that carry two transgenic markers mNeon fused to histone H2B to label the nucleus and tdTomato to label the parasite cytoplasm (*C.p*. H2B-mN-tdTom). The intestines of infected mice were sectioned and imaged as described. The number and shape of nuclei labeled in green, along with the shape and size of the cytoplasm labeled in red, allowed us to stage parasites into asexual meronts, male gamonts, and female gametes (7). Parasites were distributed along the villus axis without a discernable stage-dependent pattern (Fig. 3F), and parasites of varied stages could be regularly seen in neighboring cells (Fig. 3F, i, ii, and iii insets at different points along the villus axis display parasites of different stages adjacent to one another). To ask how oocysts are localized, we stained intestinal sections with Crypt-a-Glo, an antibody that specifically labels oocysts, and Sporo-Glo, which recognizes all parasite stages (Fig. 3G). We found that the position of Crypt-a-Glo and Sporo-Glo-positive parasites along the villus was not significantly different (Fig. 3H), showing that oocysts, like all other stages, are distributed closer to the villus tip. We also used the scRNA-seq data set, which contains host as well as parasite sequences, and we used alignment to the *C. parvum* genome to identify infected cells. Using life cycle stage-specific gene signatures from the *Cryptosporidium* single-cell atlas [VEuPathDB, release 46 (39)], we next determined the life cycle stage of each parasite and painted this information onto the enterocyte UMAP (Fig. 3I). Next, we compared infected with uninfected bystander cells and found them to have higher pseudotime values (Fig. 3J), demonstrating that infected cells were more mature consistent with a position higher on the villus as shown by two-photon imaging (Fig. 3A through E). We also painted and plotted the life cycle stage onto UMAP and pseudotime. We found the relative position of infected cells along the villus axis to be comparable regardless of the parasite life stage (Fig. 3J). We conclude that the presence of parasite-infected cells near the villus top is independent of the life cycle stage.

### Increased epithelial proliferation and survival correlate with increased parasite burden

**Fig 4:**
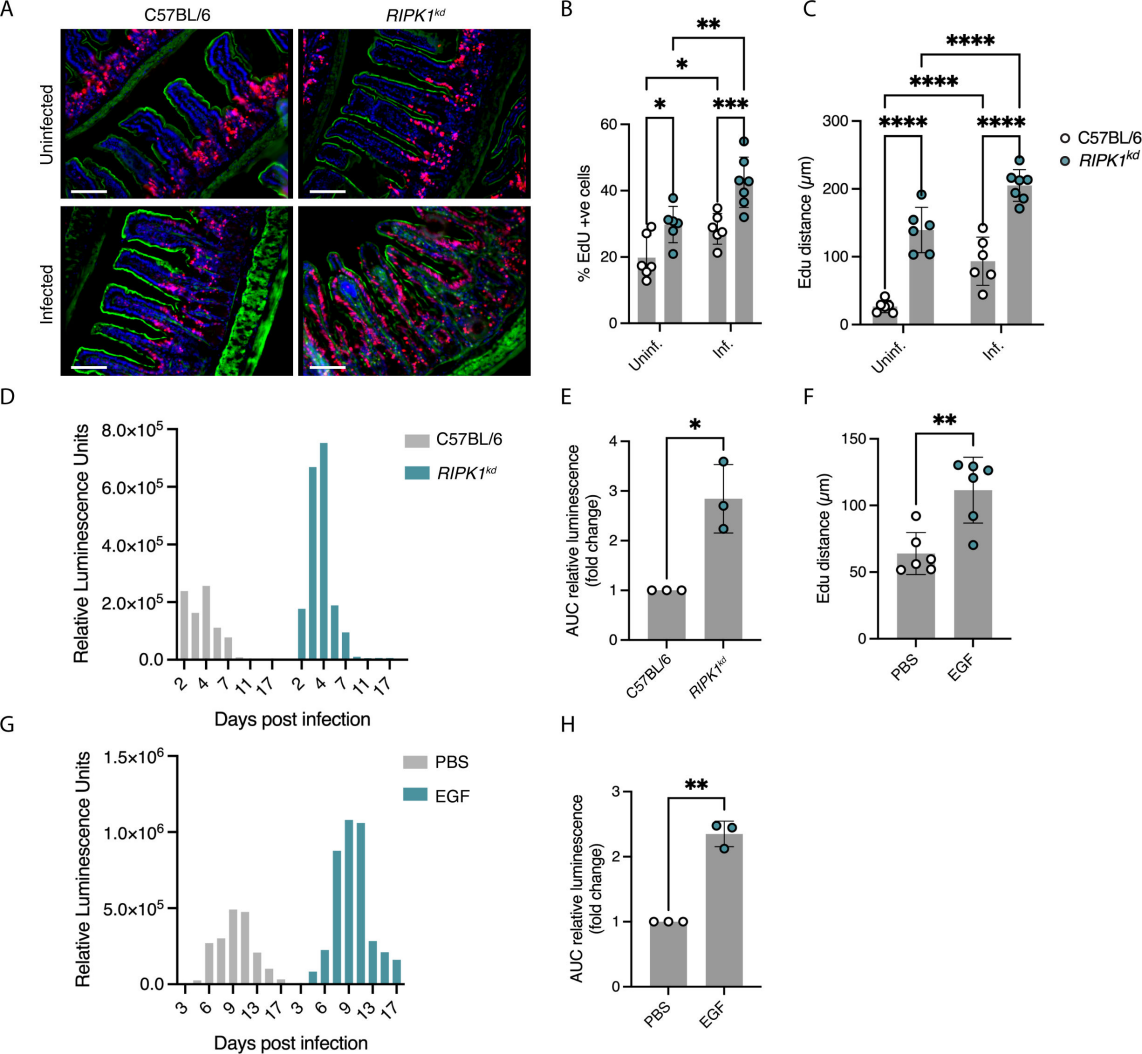
Increased parasite burdens correlate with enhanced cell proliferation and migration in *RIPK1^kd^* and epidermal growth factor (EGF)-treated mice. (**A**) Representative micrographs of intestinal sections of C57BL/6 and *RIPK1^kd^* and infected and uninfected mice. EdU labeling in red, phalloidin in green, and Hoechst-stained IEC nuclei in blue; scale bar, 100 µm. *RIPK1^kd^* mice display higher percentages of EdU-positive cells (**B**) and greater EdU migration distances (**C**). Each data point represents a mouse for which quantification from three images from a section was averaged. Three to four mice for each of two independent experiments. *P < 0.05, **P < 0.01, ***P < 0.001, and ****P < 0.0001, multiple comparison two-way ANOVA. Error bars show SD. (D) Fecal nLuc assay comparing C57BL/6 and *RIPK1^kd^* mice, from feces pooled from three to four mice per group; one representative example of three independent experiments shown (all experiments can be found in Fig. S3A). (**E**) Fold change area under curve of nLuc values normalized to C57BL/6. Each data point represents one independent experiment. Feces pooled from three to four mice in each of three independent experiments. *P < 0.05, Welch’s *t*-test. Error bars show SD. (**F**) EdU front migration distance in mice injected with PBS or EGF. Each data point represents a mouse for which quantification of three images was averaged. Three to four mice for each of two independent experiments. **P < 0.01, *t*-test. Error bars show SD. (**G**) Fecal nLuc assay of PBS or EGF-treated *Ifnγ^−/−^* mice, from feces pooled from three to four mice per group; one representative example of three independent experiments shown (all experiments can be found in Fig. S3B). (**H**) Fold change area under curve of nLuc values normalized to PBS. Each data point represents one of three independent experiments with feces from three to four mice pooled per experiment. **P < 0.01, Welch’s *t*-test. Error bars show SD.

Enhanced cell loss may contribute to parasite clearance. Therefore, we next asked whether parasite burden would be impacted by experimentally enhancing epithelial cell survival. Receptor-interacting serine/threonine-protein kinase 1 (RIPK1) acts in the pathway that leads to necroptotic cell death (40), and loss of kinase function promotes cell survival (40, 41). We, thus, tested RIPK1 kinase dead (*Ripk1^kd^*) mice as a model of reduced intestinal cell death. Crypt cell proliferation and epithelial migration rates at homeostasis have not previously been analyzed in these mice; therefore, we first injected naïve *RIPK1^kd^* mice with EdU to quantify these variables. The numbers of EdU-positive cells and their migration distances in *RIPK1^kd^* mice were significantly increased when compared to the C57BL/6 wild type (Fig. 4A through C). When *RIPK1^kd^* mice were infected with ma*Cp*, cell proliferation and EdU front migration were further enhanced (Fig. 4A through C). We also measured parasite burden by the fecal luciferase assay. Notably, *RIPK1^kd^* mice displayed increased parasite burden, with infection peaking at day 4 about 3.5× higher than observed in C57BL/6 wild-type control mice (Fig. 4D; Fig. S3A), and delayed parasite clearance by 10 days (clearance defined as relative luminescence reading of <1,000 units). We calculated the area under the curve across three independent experiments and found the burden increase to be consistent and significant (*P* < 0.05; Fig. 4E).

To reinforce our results, we looked for an alternative method to manipulate epithelial turnover and, therefore, tested the effect of EGF on parasite control. EGF is a mitotic factor that binds to receptors to promote crypt cell proliferation and regeneration (37), and intraperitoneal injection of mice with EGF has previously been used to enhance epithelial stem cell proliferation and cell migration (30). In our transcriptional analysis, we noted that infection was associated with an increase in expression of the EGF receptor, Egfr (Fig. S2A and D). EGF reduces IEC shedding through both apoptotic and non-apoptotic pathways (42). We reproduced this effect in our study. EdU assays showed that EGF treatment leads to significantly enhanced epithelial migration (*P* < 0.01; Fig. 4F; Fig. S3B). Next, *Ifnγ^−/−^* mice were infected with *C. parvum* expressing the nLuc reporter and injected intraperitoneally with PBS or 10 µg EGF on days −1, 2, and 5 of infection and every fifth day thereafter. Consistent with our findings in *Ripk1^kd^* mice, mice that received EGF displayed higher peak parasite burden (Fig. 4G) and an approximately twofold higher overall infection when measuring the area under the curve (*P* < 0.01; Fig. 4H). These mice also experienced delayed clearance, when compared to PBS-treated mice. Taken together, the *RIPK1^kd^* and EGF models suggest that enhanced epithelial proliferation and reduced IEC death correlate with increased parasite burden.

### *Cryptosporidium* infection confers enhanced resistance to induced apoptosis of the host cell

**Fig 5:**
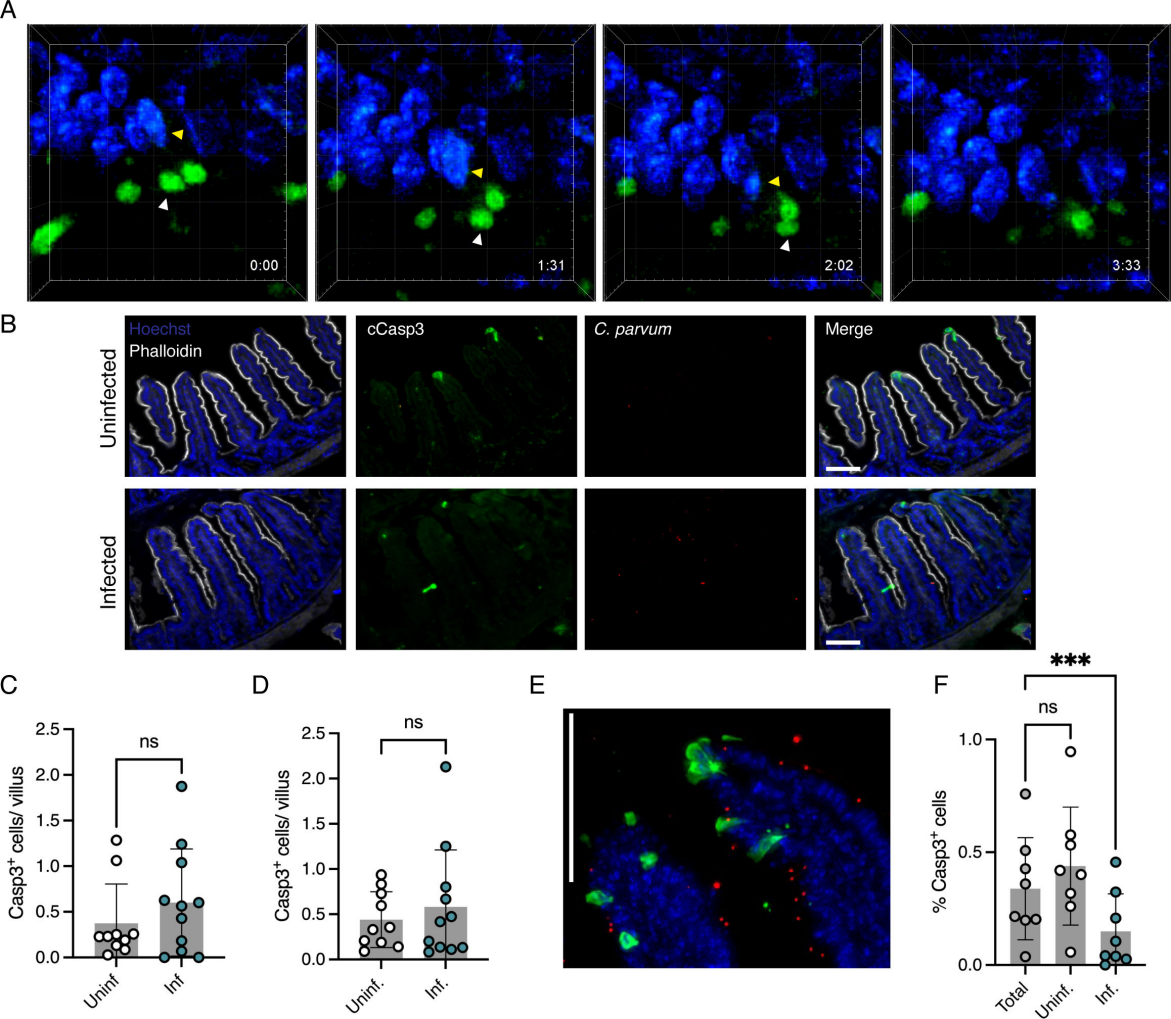
cCasp3 measurement in *Cryptosporidium*-infected mice. (**A**) Stills from two-photon intravital imaging of *C. parvum* mNeon (green)-infected intestines from Movie S1 of infected extruding cell. The parasite of the extruding cell remains associated with the host cell as it detaches from the monolayer. Hoechst-stained IEC nuclei in blue. Yellow arrow, nucleus of expelled cell; white arrow, parasite in that cell. Grid square, 10 µm, min:s. (**B**) Immunofluorescent-stained intestinal sections from uninfected and *C. parvum*-infected *Ifnγ^−/−^* mice. Hoechst-stained IEC nuclei in blue, *C. parvum* mNeon in red, cCasp3 in green, and phalloidin in gray. Scale bar, 100 µm. (**C, D**) Quantification of cCasp3^+^ cells from intestinal sections of *Ifnγ^−/−^* (**C**) and C57BL/6 (**D**) mice shows no significant (ns) difference, *t*-test. *n* = 3 experiments, three to four mice/group. Error bars show SD. (E) Immunofluorescent-stained section of infected intestine showing no overlap between cCasp3 (green) and parasite (red; Cp lactate dehydrogenase antibody) signal. An area with high numbers of cCasp3^+^ cells was specifically chosen to highlight the lack of overlap. Hoechst-stained IEC nuclei in blue. Scale bar, 100 µm. (**F**) Quantification from microscopy of percentage of total, uninfected, and infected cCasp3-stained cells. *n* = 2, four mice per experiment. ns, not significant, ***P < 0.001, one-way ANOVA. Error bars show SD.

The observation that enhancing cell survival correlated with increased parasite burden suggested that host cell death may contribute to parasite clearance. We, therefore, used intravital imaging to visualize the fate of infected cells and determine whether they are lost from the epithelium. Mice were infected with *C. parvum* constitutively expressing a neon fluorescent protein. At the peak of infection, these mice were gavaged with loperamide to reduce peristaltic movement, anesthetized, and injected retroorbitally with Hoechst DNA dye. The ileal loop was then isolated, and a small section was dissected longitudinally to expose the luminal surface. To ensure continued blood flow, the exposed tissue was kept intact with the rest of the ileum. Loperamide and indomethacin were applied topically to further slow intestinal movement and aid continuous imaging. Figure 5A (Movie S1) shows an example of the loss of an infected cell recorded as part of this experiment. The infected cell denoted by the yellow arrow is extruded and becomes detached from the epithelium. This is a rapid process with the cell and the parasite it contains disappearing from view in 3 min. Extrusion, thus, may be one form of host cell death that contributes to parasite clearance.

Overall, the mechanisms of host cell death during *Cryptosporidium* infection are not well understood, and while multiple studies have focused on apoptosis specifically, they have come to at times contradictory conclusions. Several *in vitro* studies have found infection to trigger apoptosis (43–45) while others suggested that infection blocks apoptosis (46) or blocks it in a stage-dependent manner (47–49). Here, we studied apoptosis during *Cryptosporidium* infection *in vivo* using reporter parasites. Intestinal sections prepared from infected and uninfected mice were stained with an antibody to the cleaved form of caspase 3 (cCasp3), a widely used cellular marker of apoptosis (9). We found that the numbers of cCasp3-positive cells were indistinguishable between infected and uninfected mice (Fig. 5B and C), and this finding was consistent in *Ifnγ^−/−^* mice infected with *C. parvum* mNeon (Fig. 5C) and in C57BL/6 wild type infected with ma*Cp* mCherry (Fig. 5D). As there are typically many more uninfected than infected cells in our samples, we considered that bystander cells might mask enhanced apoptosis in infected cells. We, therefore, scored apoptosis (cCasp3 staining) in infected (parasite fluorescence) and uninfected enterocytes. We were initially surprised to find that parasites residing in cCasp3^+^ cells were actually rare (Fig. 5E). Quantification across multiple experiments demonstrated the significance of this observation (Fig. 5F) with a greater than twofold decrease in the percentage of infected cCasp3^+^ cells compared to the percentage of cCasp3^+^ cells across the total population (*P* < 0.001). This implies that *Cryptosporidium* avoids infecting apoptotic cells or that it may be able to modulate apoptosis in the cells it already resides in.

**Fig 6:**
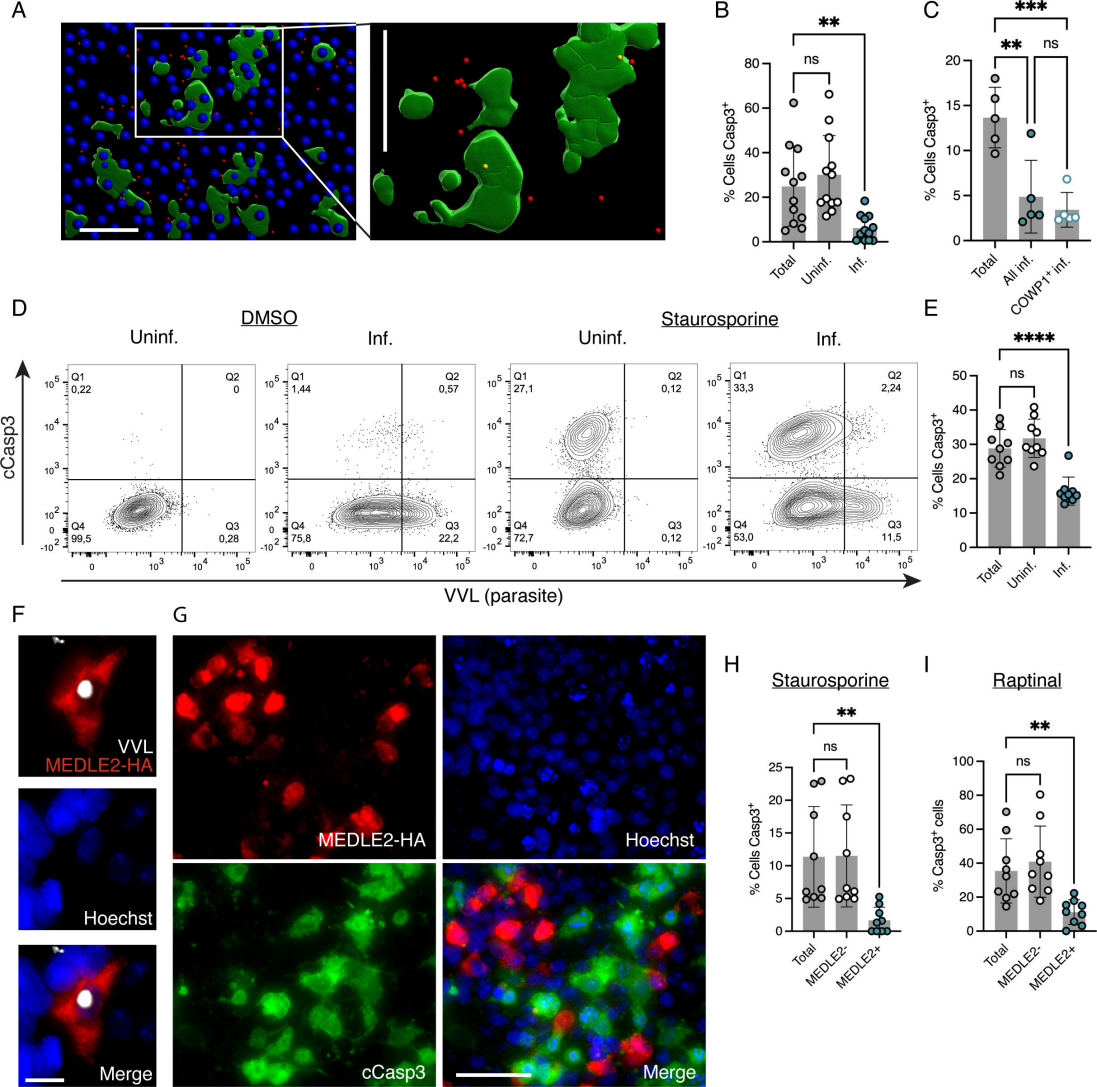
*In vitro* cCasp3 staining demonstrates that parasite-infected cells are resistant to induced apoptosis. (**A**) Representative Imaris rendered image of staurosporine-treated, *C. parvum*-infected HCT-8 cells. Blue spots are Hoechst, green surfaces are cCasp3, and red spots are parasites. The inset shows an example of two parasites within cCasp3^+^ cells (yellow spots). Note that most parasites do not overlap with Casp3 signal. Scale bars, 50 µm. (**B**) Quantification from microscopy of the percentage of total, uninfected, and infected cCasp3-stained cells from infected, staurosporine-treated cultures. Twenty-four hours post-infection. Each data point represents the mean of three fields of view per well of infected HCT-8 cells. Measurements were performed with triplicate wells in four independent experiments. One-way ANOVA; ns, not significant, **P < 0.01. Error bars show SD. (**C**) Quantification from microscopy of staurosporine-treated cultures. Percentage of total cCasp3 stained compared to all infected (cells containing both COWP1^+^ and COWP1^−^ parasites) and COWP1^+^-infected (COWP1^+^ only), cCasp3-stained cells. Experiments performed with triplicate wells as described in panel **B**; two independent experiments. Two-way ANOVA; ns, not significant, **P < 0.01, ***P < 0.001. Error bars show SD. (**D**) Representative flow plots from dimethyl sulfoxide or staurosporine-treated, uninfected, or *C. parvum*-infected HCT-8s. *n* = 3. (**E**) Percentage of total, uninfected, and infected cCasp3^+^ cells calculated by flow cytometry of staurosporine-treated cultures 24 hours post-infection. Each data point represents one well of infected cells. Experiments performed three independent times with three wells each. One-way ANOVA; ns, not significant, ****P < 0.0001. Error bars show SD. (**F**) Representative image of MEDLE2-HA (red)-infected HCT-8. VVL-stained parasites in gray and Hoechst-stained cell nuclei in blue. Note that HA staining clearly defines infected host cell, and several uninfected HA- and VVL-negative cells can also be seen. Scale bar, 10 µm. (**G**) Immunofluorescent staining of staurosporine-treated MEDLE2-HA-infected HCT-8 cultures. Hoechst-stained cell nuclei in blue, HA in red, and cCasp3 in green. Scale bar, 50 µm. (H, I) Percentage of cCasp3^+^ cells from infected, staurosporine (**H**)- or raptinal (**I**)-treated cultures comparing the total percentage of cells cCasp3^+^ to those that are cCasp3^+^ and MEDLE2-negative or positive. Experiments performed with wells as described in panel **B**; three (**H**) or two (**I**) independent experiments. One-way ANOVA; ns, not significant, **P < 0.01. Error bars show SD.

To more directly investigate the relation between infection and apoptosis, we used a tissue culture system in which apoptosis can be robustly induced using the kinase inhibitor staurosporine (50, 51). HCT-8 cells, a line of colon adenocarcinoma cells commonly used to culture *Cryptosporidium*, were infected with *C. parvum*. Twenty-one hours post-infection, cultures were treated with 2 µM staurosporine for 3 hours. Cells were then fixed and stained for cCasp3, which showed that treatment with staurosporine led to a significant increase in the numbers of apoptotic cells in both infected and uninfected cultures (Fig. S4A and B). There was no significant difference in the overall number of cCasp3-positive cells depending on infection. We next used Imaris to score cells for parasite infection and cCasp3 staining (Fig. 6A) and found them inversely correlated. Infected cells were fourfold less likely to be cCasp3-positive when compared to uninfected bystander cells in the same culture (Fig. 6B). Treatment did not result in significant differences in the numbers of infected cells, suggesting that the low number of parasites in apoptotic cells was not a result of staurosporine-induced parasite death or preferential loss of infected cells (Fig. S4C). At the 24-hour time point assayed here, only asexual parasites are present in the culture (52). We, thus, repeated these experiments at 48 hours a time point when sexual stages are present (7). We used a parasite strain that expressed tdTomato under the control of the COWP1 (52) promoter, which is solely expressed in female parasites, and co-stained all parasites using *Vicia villosa* lectin (VVL) and for apoptosis using cCasp3. Again, we found infection to be protective from induced apoptosis with a similar frequency in all intracellular life cycle stages scored (Fig. 6C).

Next, we used flow cytometry to conduct imaging-independent measurements. Following infection and staurosporine treatment, cells were suspended by trypsin-EDTA treatment, fixed and permeabilized, and stained for cCasp3. Parasites were stained with VVL conjugated to biotin followed by streptavidin-APC to gate on infected cells in the far-red channel while cCasp3 was scored in the green channel, which showed robust staurosporine-mediated cCasp3 induction (Fig. 6D). The overall percentage of cCasp3^+^ cells was not significantly different between uninfected and infected cultures within the dimethyl sulfoxide or staurosporine-treated groups (Fig. S5). However, again, the percentage of infected cells that displayed cCasp3 staining upon staurosporine treatment was significantly lower than uninfected bystanders and total cells (Fig. 6E).

Lastly, we sought to validate our findings using a marker that can be unambiguously associated with parasite infection. Intracellular stages of *C. parvum* translocate the parasite factor MEDLE2 into the cytoplasm of their respective host cell (53). This requires the establishment of a translocon that becomes active approximately 4.5 hours into the 12-hour intracellular replication cycle. We used parasites in which MEDLE2 carried a HA epitope tag, and as previously reported, this resulted in strong cytoplasmic labeling of infected host cells (Fig. 6F). When treated with staurosporine, MEDLE2-HA-positive cells showed significantly lower cCasp3 staining than HA-negative cells in the same culture (Fig. 6G and H). To broaden our observation, we also used raptinal, an apoptosis inducer with a mode of action distinct from staurosporine (54). HCT-8 cultures were infected with MEDLE2-HA parasites and treated with 10 µM raptinal, 21 hours post-infection for 6 hours. As for staurosporine, in raptinal-treated cultures, MEDLE2-HA-positive cells showed significantly lower cCasp3 staining than HA-negative cells (Fig. 6I). Taken together, we conclude that in mice, infected cells are less likely to be apoptotic than uninfected cells, and in culture, infection confers resistance to induced apoptosis.

## DISCUSSION

Infection with *Cryptosporidium* is a leading cause of enteric disease (1), and cryptosporidiosis causes profuse, watery diarrhea associated with abdominal pain, nausea, and fever. The mechanisms by which *Cryptosporidium* infection causes disease are not fully understood. Currently, there is no evidence for the presence of an exotoxin, and previous studies have focused on inflammation and barrier disruption. Mediators of inflammation including substance P and prostaglandin have been suggested as drivers of disease (55, 56), and *in vitro* studies have shown an increase of enterocyte paracellular permeability upon *Cryptosporidium* infection, a mechanism known to contribute to diarrhea (57, 58). Histopathological changes of the small intestine have also been considered (reviewed in reference 55) including loss of barrier integrity through cell loss and tissue damage. In the current study, we demonstrate significant structural and physiological changes with infection through imaging, molecular assays, and transcriptional profiling. We documented elevated loss of cells from the epithelium and increased epithelial proliferation. Enhanced proliferation in the crypts has been associated with intestinal injury (59), and a reduction of mature IECs has been observed in other enteric infections as well as gut-associated inflammatory diseases. For example, poly(I:C) treatment of mice, a model that mimics viral gastroenteritis, causes enhanced stem cell proliferation, significant loss of mature IECs, and a transcriptional switch of the epithelial cells to a reparative state with reduced absorptive and metabolic function (60, 61). Similarly, a reversion to a fetal-like program in IECs has been noted in several intestinal disease models including dextran sodium sulfate-induced colitis (36) and nematode infection (35). Our work demonstrates an overall reduction in maturity of the epithelial barrier during *Cryptosporidium* infection, with a loss of mature cells and a transcriptional shift to proportionally more stem and villus base cells. This is likely a consequence of proportionally more infection of the mature cells higher up the villus leading to their lysis upon parasite egress and the increased proliferation of cells in the stem cell compartment. Enterocytes throughout the villus are not uniform in function but instead have distinct capacities for nutrient uptake. For example, whereas enterocytes at the base of the villus display antimicrobial expression patterns, those at the middle and tip of the villus are those most responsible for carbohydrate and lipid uptake (34, 62). It is, thus, conceivable that *Cryptosporidium* infection negatively impacts the absorptive capacity of the small intestine and, thus, contributes to malnutrition. Malnutrition is not always a simple reflection of food shortage but often a syndromic consequence of enteric enteropathy dysfunction (EED) caused by constant exposure to certain pathogens, which can lead to villus blunting, atrophy, lengthened crypts, and changed intestinal permeability (63, 64). The severity of EED has also been associated with a reduction in the maturity of the intestinal epithelium (65). Acute *Cryptosporidium* infection in children can cause malnutrition due to nutrient loss in diarrhea as well as through EED leading to lasting growth impairment and developmental stunting (3, 66, 67). This may suggest that the structural impact of infection and its effects on development outlast acute cryptosporidiosis. Consistently, growth stunting is frequently observed in the context of non-diarrheal cryptosporidiosis (66, 67), suggesting lasting parasite-mediated changes to the intestine beyond acute diarrhea.

The premature death of the host cell is detrimental to intracellular pathogens, as it prevents the completion of development and, in the case of *Cryptosporidium,* the assembly and release of a new generation of merozoites to carry the infection to the next cell. Our finding that parasite-infected cells are rarely apoptotic and that infected cells are resistant to induced apoptosis suggests that *Cryptosporidium* might interfere with the death of its host cell. The literature is inconsistent on the role of apoptosis in *Cryptosporidium* infection, and this may reflect differences in the assay. Ojcius et al. used propidium iodide uptake in HCT-8s, and Chen et al. quantified nuclear condensation of biliary epithelial cells as a proxy and suggested an overall increase of apoptotic cells in infected cultures (43, 45). Mele et al. did not detect an increased propidium iodide uptake (49) but noted enhanced Annexin V staining. We use cCasp3 as a marker of apoptosis in imaging and flow cytometry to measure the relationship between apoptosis and *Cryptosporidium* infection on a single-cell basis. Transgenic parasites expressing MEDLE2-HA provide a marker to unambiguously identify infected cells. Overall, our experiments suggest that infection protects from apoptosis. Liu and colleagues observed increased transcription for host genes encoding antiapoptotic proteins during infection (47, 48) supporting the hypothesis that *Cryptosporidium* may modulate host cell apoptosis.

The mechanism that may underlie *Cryptosporidium*-mediated interference with apoptosis is unknown. Multiple molecular mechanisms to block cell death have been described for viral and bacterial pathogens including direct inhibition of caspases, downregulation of proapoptotic genes, blocking of cell death receptors, and interference with mitochondrial pore formation and cytochrome c release (68–70). Protozoan parasites have also been shown to interfere with apoptosis, displaying examples of both pro- and antiapoptotic activity (71). Among apicomplexans, antiapoptotic modulation of the host cell has been studied most intensively in *Theileria parva*. This parasite transforms and immortalizes its host lymphocytes allowing it to proliferate massively along with its host cell, without the risk of extracellular exposure. Mechanistically, this has been linked to constitutive NF-κB activation causing lymphocyte proliferation and resistance to apoptosis (72, 73). Several parasite-secreted proteins have been associated with this phenomenon, including proteins exposed to the schizont surface that are hypothesized to mediate NF-κB activation through the manipulation of the host cytoskeleton (74). *Theileria* can also export proteins to the host nucleus (75) and cytoplasm (76, 77) altering host gene expression and metabolic state to enhance survival. Similarly, the apicomplexan parasite *Toxoplasma gondii* is resistant to apoptosis in non-lymphocyte cell types (78) and inhibits host cell apoptosis through interference in a variety of host cellular pathways (79, 80) such as caspase inactivation and NF-κB activation (81, 82). Again, translocated parasite proteins, such as the dense granule protein GRA15, have been linked to the promotion of cell survival (83). It appears likely that the resistance of *Cryptosporidium*-infected cells to apoptosis is similarly dependent on parasite factors exported to the host cell. *Cryptosporidium* translocates the protein MEDLE2 into the cytoplasm of the host cell (53) in a fashion that bears resemblance to export mechanisms across the parasitophorous vacuole described for *Plasmodium* and *Toxoplasma* (84, 85). Additional candidates for antiapoptotic effectors may be found among the roughly 150 proteins recently identified as secreted during invasion (86). Several of these have been documented to translocate to the host cell or parasite interface with domains facing into the host cytoplasm (87). Further identification and characterization of exported parasite factors will be key to understanding the mechanisms behind apoptotic interference by the parasite.

The observation that parasite infection protects cells from apoptosis may seem at odds with the finding that *Cryptosporidium* infection results in enhanced loss of enterocytes documented by EdU labeling and measurement of host DNA in feces. However, there are multiple mechanisms of cell loss that are independent of apoptosis. Host cell lysis due to parasite egress is one, and the high number of infected cells observed in the tissue (Fig. 3A, B, and F) combined with the fast 12-hour parasite replication cycle (7) could indeed inflict significant loss. We show that cells are also lost through caspase 3-independent mechanisms, which may include necroptosis or pyroptosis. This is consistent with the finding that ablating elements of the inflammasome such as *Nlrp6* and *Caspase 1/11* enhances parasite survival (22, 88); however, this was previously linked to IL-18 secretion that is important for IFN-γ production rather than pyroptosis. We also show that loss of RIPK1 kinase activity, which contributes to necroptotic signaling (40), led to enhanced parasite burden. Increased parasite burden in EGF-treated and *RIPK1^kd^* mice may reflect increased availability of host cells or point to a role of necroptosis or other non-apoptotic means of cell death in limiting parasite survival. This agrees with recent work by Wong et al. who demonstrated in enteroids reduced TNF-induced cell death upon the inactivation of RIPK1 kinase (89). Beyond programmed cell death leading to IEC extrusion, accelerated cell proliferation and migration in the crypts result in crowding at the villus tips leading to an increase in the expulsion of cells from the epithelial layer (90, 91). Again, such cells would be missed by cCasp3 staining (91). Extrusion of living enterocytes is part of normal homeostatic turnover and appears elevated during *Cryptosporidium* infection. Enhanced extrusion is likely a means of eliminating infected cells, even when the parasite antagonizes cell death. Future studies may shed light on how infected cells are recognized by the host to initiate extrusion. Extrusion of infected IECs as a means of controlling infection has been shown in *Salmonella* infection, where infected epithelial cells undergo non-lytic, inflammasome-driven expulsion causing restriction of the bacteria (92, 93). A recent study demonstrated that inhibition of apoptosis in the intestine through epithelial cell-specific knockout of caspase 3 and 7 led to enhanced levels of non-apoptotic cell extrusion from the mucosal barrier (94). A similar mechanism of enhanced non-apoptotic cell extrusion may be active during *Cryptosporidium* infection to compensate for the reduction in apoptosis of infected cells. It would be interesting to understand the fate of infected cells and whether the parasites within them are able to persist and potentially even egress or be shed as oocysts or whether these parasites are always lost. Acquisition of *Cryptosporidium* antigen by dendritic cells is important to elicit an immune response (95, 96). However, how antigen is acquired is not yet fully understood. Uptake from extruding infected cells may be one means of doing so.

It is critical for the host to maintain a functional mucosal barrier. We show that *Cryptosporidium* infection causes significant changes in the structure and physiology of the gut that may come with lasting consequences. The survival of infected cells appears to be a key battleground between host and parasite. The host can marshal multiple ways to rid itself of infected cells, while the parasite appears capable of antagonizing at least some of them. Understanding the underpinning molecular mechanism not only is critical to combating this parasite infection and its sequelae but also may deepen our insight into the role of cell turnover in response to disease and injury in the gut in general.

## MATERIALS AND METHODS

### Mouse models of infection

*Ifnγ^−/−^* (stock no.: 002287; RRID: IMSR_JAX:002287) and C57BL/6 (stock no.: 000664) were purchased from Jackson Laboratory. *Ripk1^kd^* (97) and *Ifnγ^−/−^* mice were maintained as a breeding colony at the University of Pennsylvania. Mice ranged from 4 to 10 weeks and were age- and sex-matched within each experiment. For EGF experiments, *Ifnγ^−/−^* mice were injected intraperitoneally (30) with PBS or 0.5 mg/kg EGF (PeproTech) in PBS on day −1, day 2, day 5, and every 5 days thereafter.

### Parasite strains

*C. parvum* oocysts were purchased from Bunchgrass Farms. Transgenic parasites were selected and propagated in 4–10-week-old *Ifnγ^−/−^* mice. Parasites expressing mNeon and COWP1-tdTom (52) and MEDLE2_HA (53) and mouse-adapted *C. parvum* (ma*Cp*) (23) have been described previously. *Ifnγ^−/−^* mice were infected with 10,000 oocysts and C57BL/6 with 100,000 oocysts.

### Plasmid construction and parasite transgenesis

The H2B-mNeon tdTomato plasmid was adapted from reference 7 to express tdTomato in the place of the mScarlet fluorescent protein. mScarlet was removed using primers 1 and 2, and tdTomato was inserted by Gibson assembly using primers 3 and 4 (Table S1). Transgenic parasites were derived as previously described (31).

### Measuring parasite burden by NanoLuc luciferase assay

Twenty milligrams of fecal material was suspended in NanoLuc luciferase lysis buffer and mixed 1:1 with NanoLuc luciferase substrate/NanoLuc luciferase assay buffer (1:50) in a white-bottom 96-well plate. Relative luminescence was read using a Promega GloMax Plate Reader. Feces for each cage (group) was pooled, as previous work showed that mice within the same cage show similar levels of infection (98). Experiments were repeated at least three independent times to calculate the area under the curve.

### qPCR analysis

gDNA was extracted from 100 mg of fecal material using the Quick DNA Fecal/Soil Microbe Kit (Zymo Research). Mouse gDNA was amplified using mouse 18S-specific primers 5 and 6 (Table S1). SYBR Green Mastermix (Thermo Fisher) was used to perform qPCR and run with 3 mM Mg^2+^ in white 384-well plates (Greiner Bio-One) on a ViiA 7 Real-Time System (Thermo Fisher) using the following conditions: 2 min at 50°C, 10 min at 95°C, and 50 cycles of 15 s at 95°C and 1 min at 60°C.

### Histological analyses

To prepare intestinal sections, mice were euthanized, and the ileum was removed, flushed with PBS, cut open longitudinally, and “Swiss-rolled.” Samples were fixed overnight at 4°C in 10% neutral buffered formalin (Thermo Fisher) followed by 30% sucrose overnight. Tissue was frozen in OCT solution (Sakura Finetek) and cryosectioned. For immunostaining, sections were thawed and incubated in PBS-0.1% Triton-1% bovine serum albumin (BSA) (hydration buffer) followed by blocking in PBS-0.1% Triton-10% BSA for 45 min each. Antibody staining was carried out in a hydration buffer. Slides were mounted with Fluoro-Gel mounting medium (Thermo Fisher). EdU assays were performed as described (25) by injection with 30 mg/kg EdU (Life Technologies) or PBS 2, 24, or 60 hours before euthanizing. Slides were stained using the Click-It EdU Imaging Kit with Alexa Fluor 594 Azide (Thermo Fisher). Hoechst or phalloidin staining was used to identify areas to image and quantify followed by the channel used to make the measurement (i.e., cCasp3 or EdU) to avoid bias. Swiss rolls were imaged in three separate areas throughout the section and averaged to derive a mean value per mouse. Imaris software was used to automate the counting and segmentation of Hoechst and EdU-positive nuclei and Crypt-a-Glo and Sporo-Glo-positive parasites. Migration distances were measured in Imaris from the top of the crypt to the highest EdU-positive cell nucleus. The coordinates for each parasite were identified from two villi per image, and their distance from the base of the villus was calculated. Image analysis was not blinded.

### Cell death assays using imaging or flow cytometry

HCT-8 cells grown on coverslips or in 24-well plates were infected with 10^6^ excysted oocysts (three coverslips per treatment). At 21 hours post-infection, wells were treated with 2 µM staurosporine (Sigma-Aldrich) for 3 hours or 10 µM raptinal (Sigma-Aldrich) for 6 hours prior to fixation in 4% paraformaldehyde (PFA; Sigma-Aldrich). For imaging, cells were permeabilized in 0.25% Triton for 12 min prior to blocking in 4% BSA and primary and secondary antibody incubation in 1% BSA. For flow cytometry, cells were suspended by 2-min incubation in 0.25% trypsin-EDTA (Thomas Scientific), resuspended and washed in Dulbecco’s modified Eagle medium (Thermo Fisher) 10% Cosmic Calf serum (Thermo Fisher), and fixed for 12 min in 4% PFA. Cells were permeabilized in eBioscience permeabilization buffer (Thermo Fisher) for 10 min, and antibodies were incubated in the same buffer. Samples were run in 1× PBS, 0.2% BSA, and 1 mM EDTA on a FACSCanto Flow Cytometry System (BD Life Sciences), and data were analyzed using FlowJo v10.8 software (BD Life Sciences).

### Immunofluorescence microscopy

Primary antibodies used were rabbit polyclonal cleaved caspase 3 antibody (Asp175 Cell Signaling Technology) at 1:400 for cultured cells or 1:200 for intestinal sections and 1:100 for flow cytometry, rat anti-HA (Sigma-Aldrich) at 1:1,000, and rabbit anti-LDH (Sigma-Aldrich) at 1:200. Secondary antibodies used were goat anti-rabbit Alexa 488, 594, or 647 (Fisher Scientific) at 1:1,000 for cultured cells, 1:500 for flow cytometry, or 1:250 for intestinal sections. The parasite-specific lectin VVL-fluorescein (Vector Laboratories) was used at 1:1,000 for cell culture imaging and 1:500 for flow cytometry, VVL-biotin (Vector Laboratories) at 1:1,000, Crypt-a-Glo-biotin (Waterborne, Inc.) at 1:20, Sporo-Glo-fluorescein 1× reagent (Waterborne, Inc.), phalloidin conjugated to fluorescein or Alexa Fluor 647 (Thermo Fisher) at 1:500, and Hoechst 33342 (Thermo Fisher) at 1:5,000 for coverslips, 1:2,000 for Swiss rolls, and 1:1,000 for flow cytometry. VVL-biotin was detected with streptavidin-APC (Thermo Fisher) at 1:500 and Crypt-a-Glo-biotin with streptavidin-594 at 1:250 (Thermo Fisher). Coverslips were mounted using Vectashield (Vector Laboratories) or Fluoro-Gel (Thermo Fisher). Images were taken using a Leica DM6000B fluorescence microscope. Images were processed and analyzed as described above for histology.

### Single-cell sequencing and analysis

scRNA-seq analyses were performed using a previously published data set (GEO repository number GSE246500) (32). Briefly, two uninfected and two ma*Cp*-infected *Ifnγ^−/−^* mice (5 days post-infection) were sacrificed, and the epithelial layer (containing IECs and intraepithelial lymphocytes) was isolated from the ileum of the small intestine. Reads were processed, and a reference genome for *C. parvum* Iowa II (VEuPathDB, release 46) was built using Cell Ranger v7.0.0. Processed reads were then aligned either to this genome or to the *Mus musculus* genome (GRCm38). To identify infected cells, reads aligning to the *C. parvum* genome were imported into R to generate a Seurat object (99). Samples were merged and filtered to remove empty droplets (<100 features), doublets (>1,200 features or nCount > 4,000), and cells in which more than 60% of gene expression was attributed to ribosomal genes (cgd2_1372, cgd2_1373, cgd3_665, cgd3_666, and cgd3_667). The remaining cells were identified as infected in the mouse genome-aligned data set.

For data aligned to the mouse genome, Seurat objects were created in R for the uninfected and infected samples (99). Samples were merged and filtered to remove empty droplets (<100 features), doublets (>10,000 UMI), and cells with >40% mitochondrial genes. Data were normalized and scaled, followed by principal component and jackstraw analyses to determine the dimensionality of the data set and UMAP dimensional reduction. Pseudotime trajectory analysis was performed using monocle3 (33, 100, 101).

Average log2 fold change expression was identified for epithelial gene markers for villus bottom landmark genes from reference 34. These genes were cross-referenced with single-cell positional clustering analysis from reference 34 whereby clusters 1 and 2 contain genes predominantly expressed at the villus base. Only those landmark genes found to also be in clusters 1 and 2 were included in the analysis (eight were excluded based on cluster positioning of >2, and two were not found in our data set).

### Whole mount tissue clearing and parasite detection and quantification

*Ifnγ^−/−^* mice were euthanized 8–10 days after infection, and tissue clearing was performed as previously described (38). Briefly, mice were perfused with PBS followed by 4% PFA. Intestines were resected and flushed with PBS. The most distal 1 cm ileal section was incubated in 4% PFA overnight at 4°C. Tissue was then incubated in 1:1 distilled H_2_O and CUBIC-L (10 wt% N-butyldiethanolamine, 10 wt% Triton X-100) rotating for 3 hours. The clearing solution was replaced with CUBIC-L and 0.5 mg/mL Hoechst 33342 (Invitrogen) and incubated at 37°C for 6 days, replacing the solution after 3 days. Samples were washed in PBS 0.01% sodium azide overnight, bisected longitudinally to expose the lumen, glued to a petri dish using Vetbond (3M), and submerged in PBS. Tissue was imaged on a SP8 multiphoton microscope (Leica Microsystems). Images were segmented, and the parasite (mNeon) and host cell (Hoechst) numbers and median parasite and host cell position were quantified using Imaris. We measured the distance of each parasite or host cell from the top of the 250-µm Z-stack using Imaris. Data were exported to RStudio, and this stack was divided (excluding the crypts) into four equal cubes, and the number of parasites was scored for each cube.

### Two-photon intravital imaging

Mice were used for live imaging at the peak of infection (8–10 days post-infection) following oral gavage with 200 µL 2 mg/mL loperamide hydrochloride (Sigma-Aldrich) to minimize peristaltic movement. Mice were anesthetized by intraperitoneal injection with a xylazine/ketamine cocktail followed by retroorbital injection with 0.5 mg Hoechst 33342 (Invitrogen) and maintained under anesthesia at 37°C with vaporized isoflurane. The ileal loop was extracted through a small incision made in the abdomen, and a 1-cm section was dissected longitudinally to expose the luminal surface. The cut edges were cauterized to minimize blood loss. Twenty micrograms per milliliter loperamide hydrochloride and 20 µg/mL indomethacin (Sigma-Aldrich) were applied topically to the luminal intestinal surface. Live and whole-mount imaging was carried out on a SP8 multiphoton microscope (Leica Microsystems) with a 20 × 1.0 NA water-dipping objective and equipped with a resonant scanner (8,000 kHz) and four external HyD detectors. The excitation wavelength of the Chameleon Vision II Ti:Sapphire laser (Coherent) was tuned to 900 nm. After imaging, mice were euthanized by CO_2_ asphyxiation. Images were analyzed with Imaris.

## SUPPLEMENTAL MATERIAL

Supporting information mbio.01720-24-s0001.docx Supplemental figures, table, and legends.

Movie S1 mbio.01720-24-s0002.mp4 Two-photon intravital imaging of *C. parvum* mNeon-infected extruding cell.

ASM does not own the copyrights to Supplemental Material that may be linked to, or accessed through, an article. The authors have granted ASM a non-exclusive, world-wide license to publish the Supplemental Material files. Please contact the corresponding author directly for reuse.
