# Supplementary material for: Cryptosporidium impacts epithelial turnover and is resistant to induced death of the host cell
Source: mBio. 2024 Jul 12;15(8):e01720-24. doi: 10.1128/mbio.01720-24 (PMC11323733; doi:10.1128/mbio.01720-24)
Supplement: Supporting information — Supplemental figures, table, and legends. [file mbio.01720-24-s0001.docx]

## Supporting Information


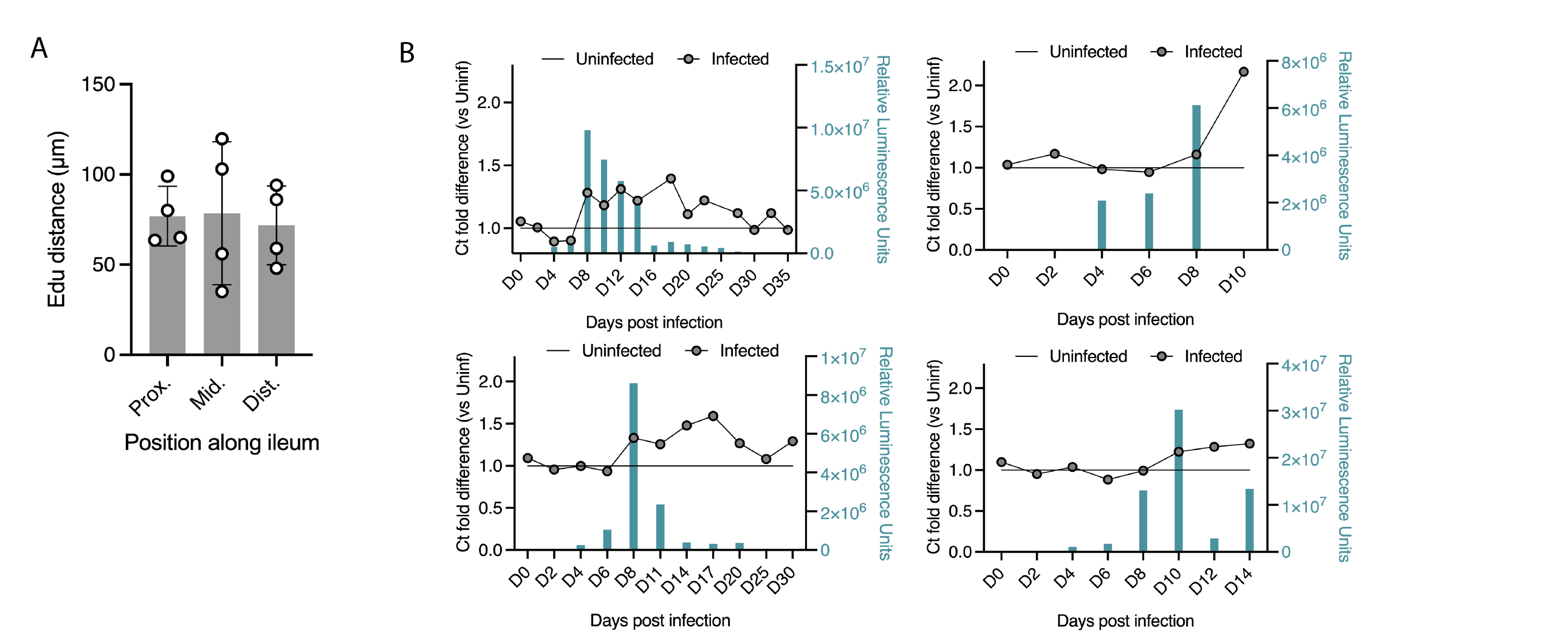


**Figure S1 Positional analysis of EdU migration and relative quantities of mouse fecal gDNA across infection. A** Distance from top of crypt to furthest EdU labelled cell quantified from proximal, middle and distal positions along the ileum. Intestinal sections from C57BL/6 mice infected with *C. parvum* mouse adapted mCherry 24 hours after EdU injection. Each data point denotes quantification of 1 mouse. No significant differences were observed using the One-Way ANOVA test. Error bars show SD. **B** Mouse gDNA extracted from feces and assessed by qPCR. Relative quantities shown as Ct (cycle threshold) fold difference comparing infected to uninfected. nLuc represents relative parasite quantities in feces plotted as bars. Each panel denotes one of 4 independent experiments, pooled feces from 3 mice per group.


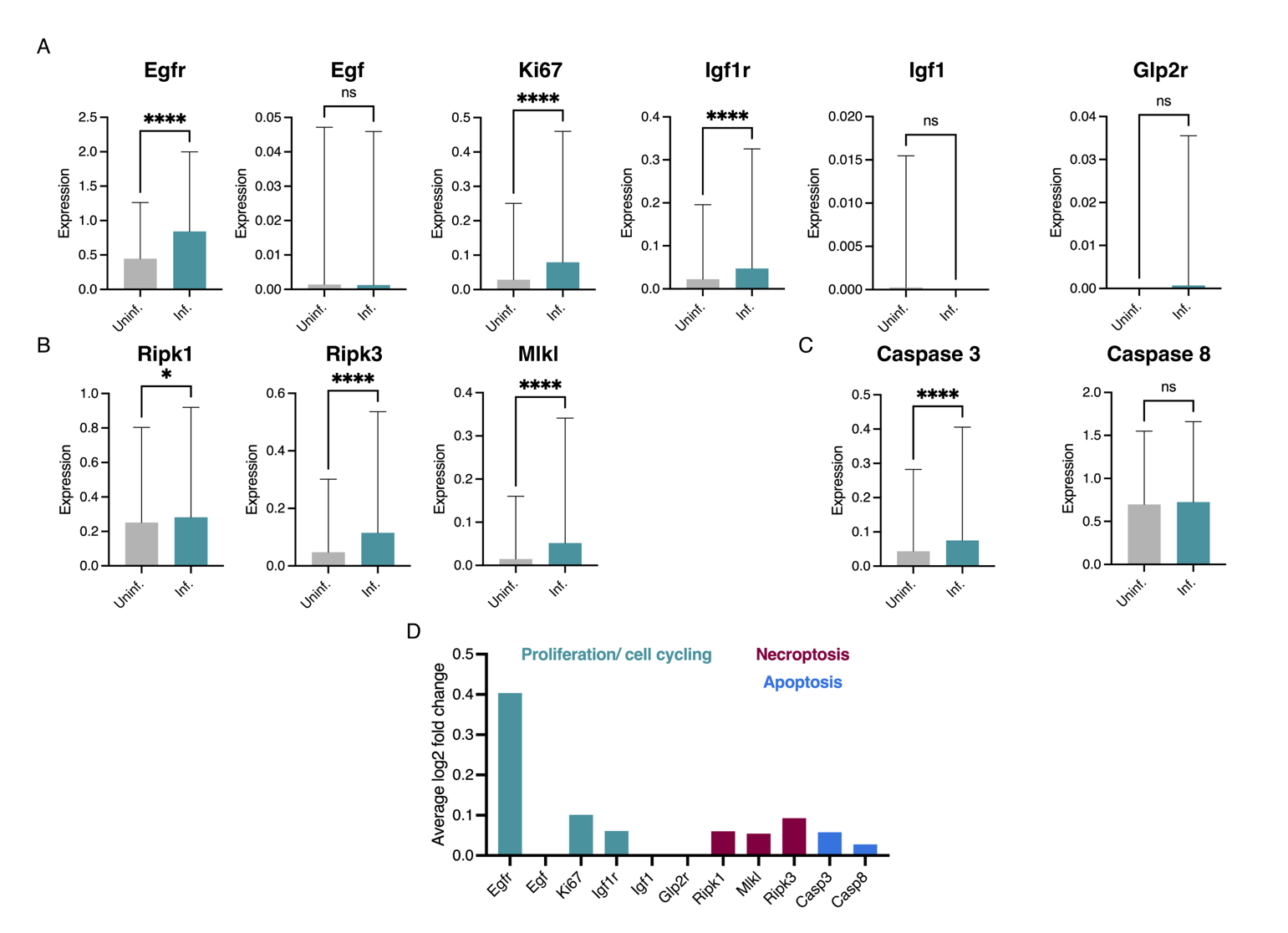


**Figure S2 Transcriptional changes of proliferation and cell death related genes during infection.** Average expression of all cells of notable gene markers for **A** cell proliferation and cycling, **B** necroptosis and **C** apoptosis from intestines of uninfected and infected *Ifnγ^-/-^* mice. 2 pooled mice per sample from one experiment. ns not significant, *p<0.05, ****p<0.0001, t-test. **D** Average log2 fold-change due to infection of maker genes displayed in A, B and C.


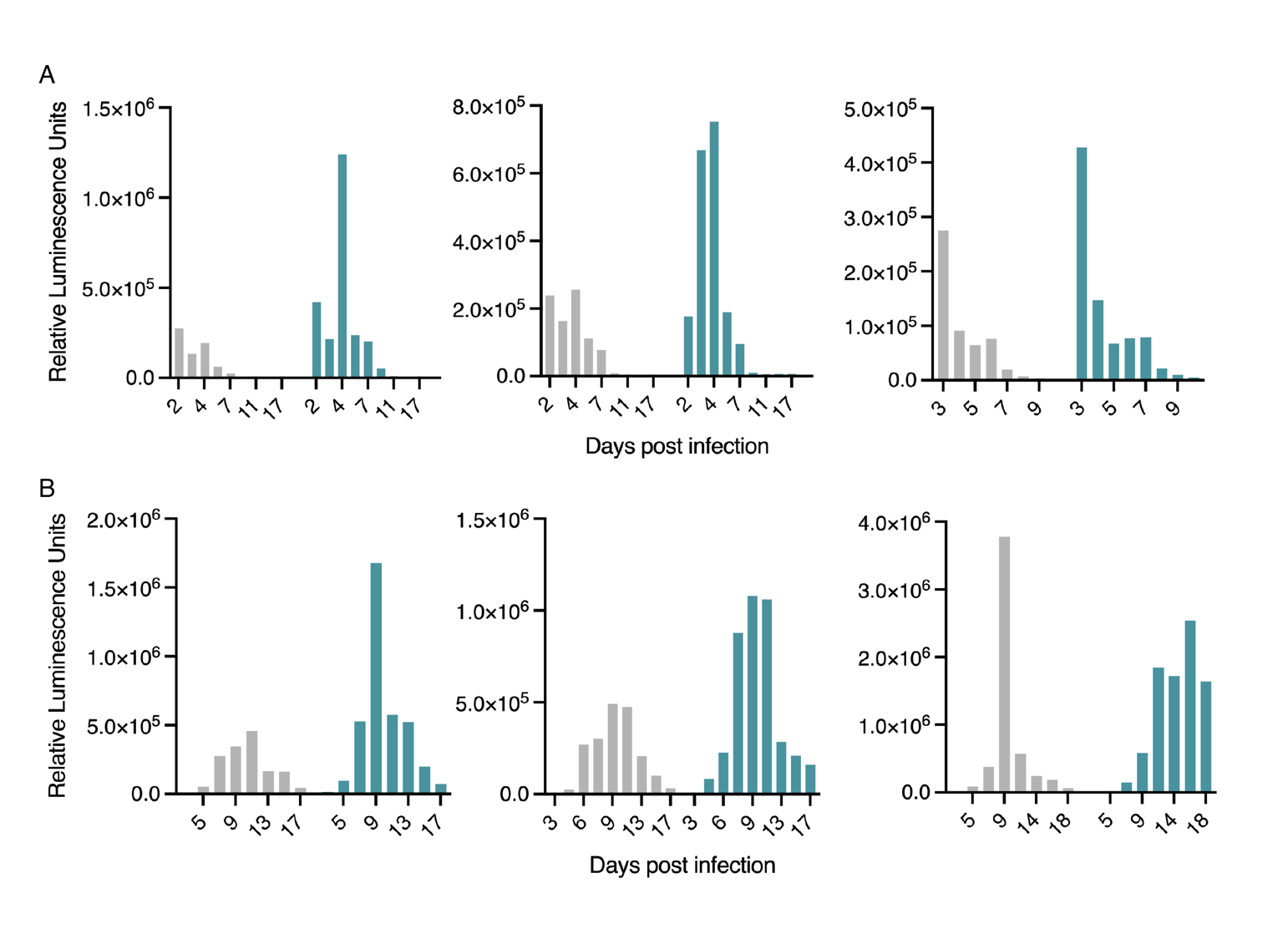


**Figure S3 nLuc assays demonstrating higher parasite burden in *RIPK1^kd^* and EGF treated mice.** Fecal nLuc assay comparing parasite burden between **A** C57BL/6 wildtype and *RIPK1^kd^* mice and **B** PBS and EGF treated *Ifnγ^-/-^* mice. Each panel denotes one of 3 independent experiments, from feces pooled from 3-4 mice per group.

**
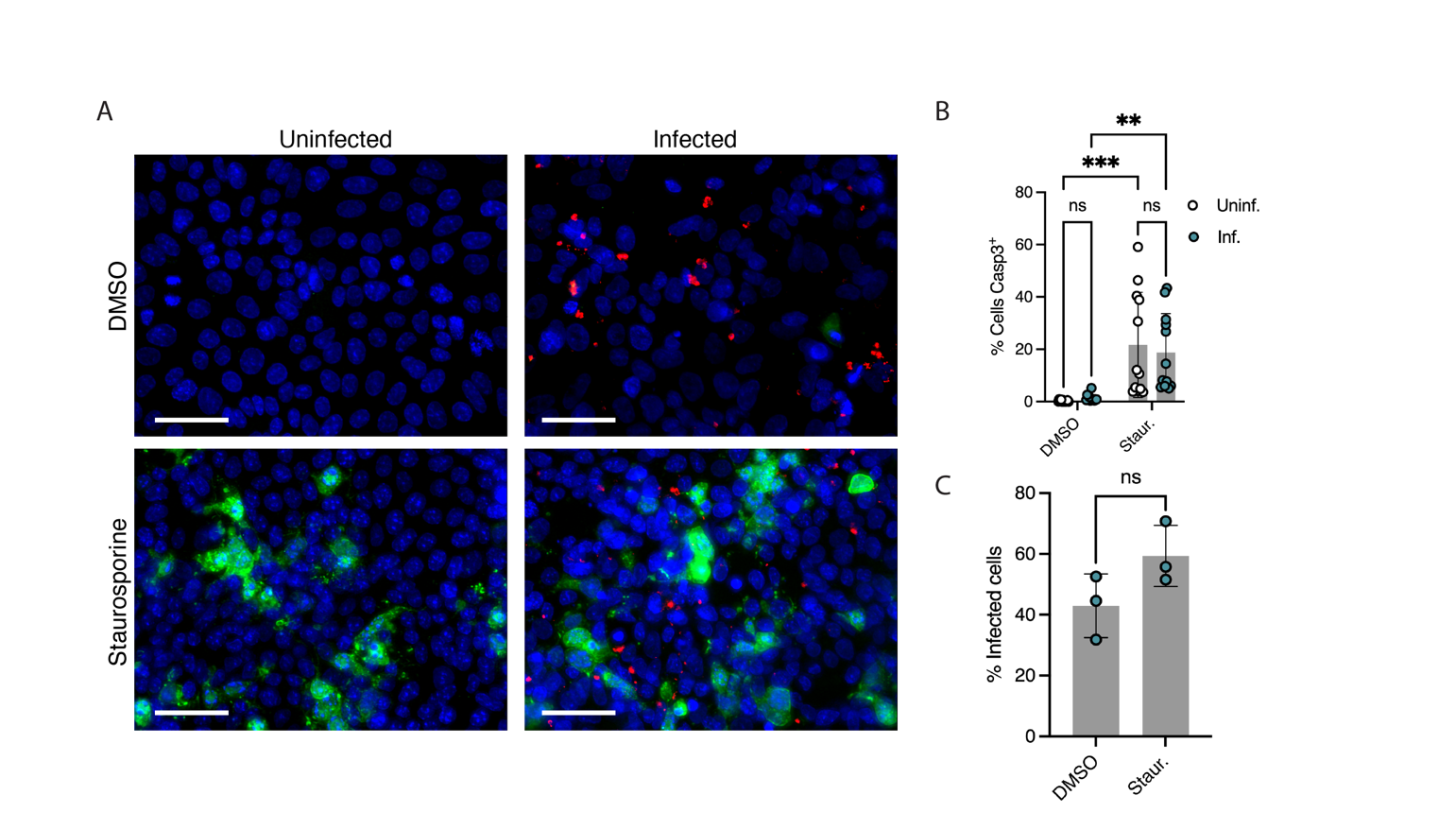
**

**Figure S4 Immunofluorescent imaging of *in vitro* staurosporine assays.A** Immunofluorescent staining of DMSO or staurosporine-treated uninfected or *C. parvum* Bunchgrass infected HCT-8 cultures. Blue is Hoechst, red is VVL (vicia villosa lectin; parasite stain), green is cCasp3. Scale bar 50 µm. **B** Percentage cCasp3^+^ cells quantified from (A). Each data point represents the mean of three fields of view per well of uninfected or infected cells. Measurements were performed with triplicate wells in 4 independent experiments. ns not significant, **p<0.01, ****p<0.0001, multiple comparison 2-way ANOVA. Error bars show SD. **C** Quantification of percentage infected cells in DMSO and staurosporine treated cultures. 1 representative of 4 independent experiments. All experiments displayed no significant difference (ns), t-test. Error bars show SD.

**
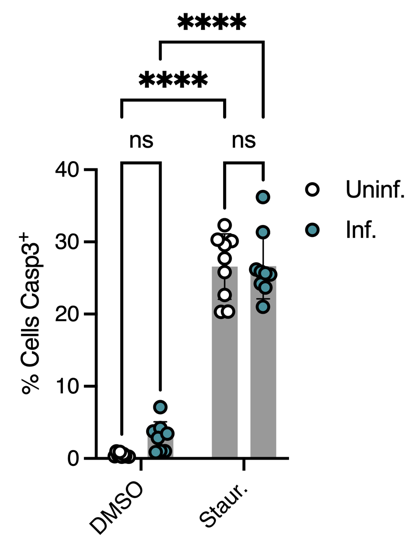
**

**Figure S5 Flow cytometry of *in vitro* staurosporine assays.**

Percentage cCasp3^+^ cells quantified by flow cytometry of DMSO or staurosporine treated HCT-8 cultures. Each data point represents a well of uninfected or infected cells. Measurements were performed with triplicate wells in 3 independent experiments. Multiple comparison 2-way ANOVA, ns not significant, ****p<0.0001,. Error bars show SD.

| **Name** | **Sequence 5’ - 3’** |
| --- | --- |
| Primer 1 | ATGGTGAGTAAGGGCGAGG |
| Primer 2 | TCACTTATACAGCTCATCCATGC |
| Primer 3 | CTGTACGGCATGGATGAGCTGTATAAGTGAcaattgGTTCGTGGCGTG |
| Primer 4 | TTTGATCACTTCCTCGCCCTTACTCACcatCGGGCCGGGGTTCTCCTC |
| Primer 5 | GTAACCCGTTGAACCCCATT |
| Primer 6 | CCATCCAATCGGTAGTAGCG |

**Table S1 Primer list.**

List of primers used in this study. Primers were used at 0.2-0.5 µM for standard PCR and 1µM for qPCR.

**Movie S1 2-photon intravital imaging of extruding, infected epithelial cell.**

2-photon intravital imaging of *C. parvum* mNeon (green) infected extruding cell. Blue is Hoechst-stained epithelial cell nuclei. Grid square 10 µm, min:sec. Several infected cells can be seen in the field of view with one extruding and becoming detached from the epithelial layer (nucleus of extruding cell denoted by yellow arrow). The parasite that is infecting this cell (white arrow) is extruded along with its host cell. The cell and the parasite disappear from the frame by 3 mins while the rest of the epithelial layer of infected and uninfected cells stays intact.
